# One-pot green synthesis of antimicrobial chitosan derivative nanocomposites to control foodborne pathogens

**DOI:** 10.1039/d1ra07070c

**Published:** 2022-01-05

**Authors:** Mahmoud H. Abu Elella, Ahmed Esmail Shalan, Magdy W. Sabaa, Riham R. Mohamed

**Affiliations:** Chemistry Department, Faculty of Science, Cairo University Giza 12613 Egypt mahmoudhussien3766@yahoo.com; Central Metallurgical Research and Development Institute (CMRDI) P. O. Box 87, Helwan Cairo 11421 Egypt ahmed.shalan@bcmaterials.net; BCMaterials, Basque Center for Materials, Applications and Nanostructures Martina Casiano, UPV/EHU Science Park, Barrio Sarriena s/n Leioa 48940 Spain

## Abstract

Food contamination by foodborne pathogens is considered a serious problem worldwide. This study aimed to show the efficacy of the one-pot green biosynthesis of nanocomposites as effective antimicrobial agents based on a water-soluble biodegradable polysaccharide and silver nitrate (AgNO_3_). Silver (Ag) nanoparticles were synthesized using different concentrations of AgNO_3_ solution (1, 2, and 3 mM) in the presence of *N*-quaternized chitosan and *N*,*N*,*N*-trimethyl chitosan chloride (TMC) as both a reducing and stabilizing agent. In addition, the structure of TMC/Ag nanocomposites was confirmed using different analytical tools including FTIR, UV-Vis, XRD, HR-TEM, FE-SEM, and EDX techniques. The FTIR spectra and UV-Vis spectra showed the main characteristic absorption peaks of Ag nanoparticles. In addition, FE-SEM images showed the formation of spherical bead-like particles on the surface of TMC. Correspondingly, the EDX spectrum showed a peak for silver, indicating the successful synthesis of Ag nanoparticles inside the TMC chains. Moreover, HR-TEM images exhibited the good distribution of Ag nanoparticles, which appeared as nano-spherical shapes. The antimicrobial activity of TMC/Ag nanocomposites was examined against three foodborne pathogens, including *Salmonella* Typhimurium as a Gram-negative bacterium, *Bacillus subtilis* as a Gram-positive bacterium and *Aspergillus fumigatus* as a fungus. The results showed that TMC/Ag nanocomposites had better antimicrobial activity compared with TMC alone and their antimicrobial activity increased with an increase in the concentration of Ag. The results confirmed that the TMC/Ag nanocomposites can be potentially used as an effective antimicrobial agent in food preservation.

## Abstract 2

This study aimed to show the efficacy of the one-pot green biosynthesis of nanocomposites as effective antimicrobial agents based on a water-soluble biodegradable polysaccharide and silver nitrate (AgNO_3_).

## Graphical abstract

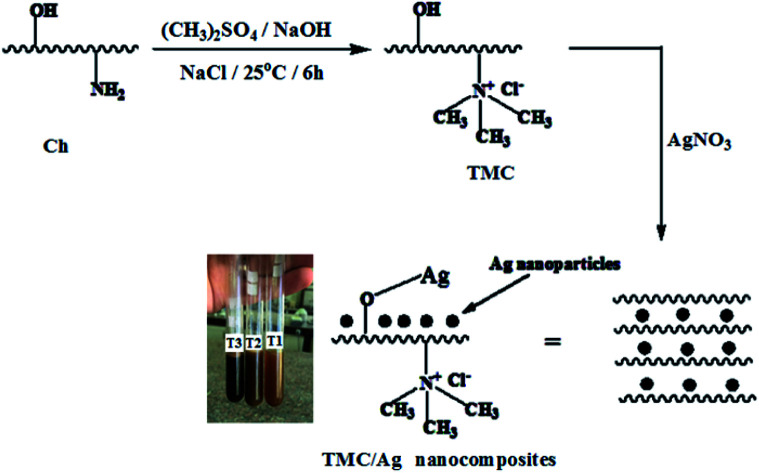

## 1. Introduction

Food products are contaminated by foodborne pathogens including *Salmonella* Typhimurium, *Bacillus subtilis*, and *Aspergillus fumigatus*. As a result of microbial contamination, the consumption of contaminated food leads to an increase in the risk of foodborne diseases, which is considered a major problem globally.^1–3^ Thus, researchers have been focused on the synthesis of effective antimicrobial agents to reduce foodborne pathogen outbreaks. Silver nanoparticles are widely used in the fabrication of new antimicrobial agents with broad spectrum activity against pathogenic microorganisms.^4,5^

Silver nanoparticles are synthesized *via* chemical reduction.^6–8^ The eco-friendly synthesis of AgNPs has been done using non-toxic reducing agents including seed extracts, living organisms, and plant leaves.^9–11^

Recently, AgNPs have been synthesized in the presence of non-toxic highly available polysaccharides such as chitosan, xanthan gum, locust bean gum, and agar as self-reducing agents.^7,12,13^

**Fig. 1:**
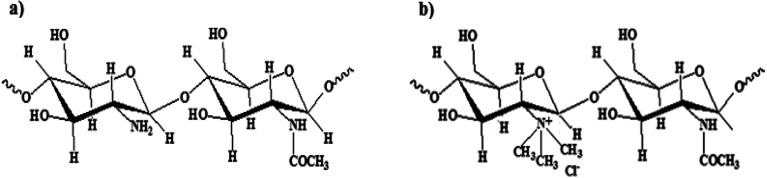
Chemical structure of (a) chitosan and (b) TMC.

Chitosan (Ch) is a modified cationic polysaccharide composed of β-(1→4)-linked copolymers of glucosamine and *N*-acetylated glucosamine units (Fig. 1a). Ch is extracted from chitin with partial alkaline hydrolysis of its *N*-acetylated units at high temperature. However, the complete deacetylation of chitin is rarely observed. Consequently, Ch has different molecular weights in the range of ≈5 × 10^4^ Da to 2 × 10^6^ Da and different degrees of deacetylation (DD) of ≈40% to 98%. Ch has substantial biological properties, such as antimicrobial, antioxidant and anti-inflammatory activities. Also, it is biodegradable, biocompatible and non-toxic.^14–18^ Ch is only dissolved in acids, and thus it has very limited applications because of its poor water solubility. Ch was chemically modified to form a water-soluble derivative, denoted as *N*-quaternized chitosan (NQC)^19–23^ (Fig. 1b), which is water-soluble over a wide pH range due to the presence of *N*-quaternary ammonium (–N^+^R_3_) moieties on its backbone, resulting in both improved water solubility and antimicrobial activities.^24^

The main purpose of the current work was to fabricate trimethyl chitosan chloride/Ag nanocomposites through green and safe biosynthesis as effective antimicrobial agents against foodborne pathogens. The structure of the TMC/Ag nanocomposites was elucidated using various techniques including FTIR, UV-Vis, XRD, HR-TEM, and FE-SEM/EDX.

In addition, the antimicrobial activity of the TMC/Ag nanocomposites was investigated against foodborne pathogens including *Salmonella* Typhimurium and *Bacillus subtilis* as Gram-negative and Gram-positive bacteria, respectively, and investigated against *Aspergillus fumigatus* as a fungus.

## 2. Experimental section

### 2.1. Chemicals

Chitosan (Ch) (DD = 90–95% and molecular weight = 16 × 10^4^ g mol^−1^) was obtained from Oxford (Maharashtra, India). AgNO_3_ was supplied by Merck (Hohenbrunn, Germany). Dimethyl sulphate ((CH_3_)_2_SO_4_) was purchased from Loba Chemie (Mumbai, India). Regenerated cellophane dialysis tubes (molecular weight cut off: 12–14 × 10^3^ g mol^−1^) were procured from Serva Electrophoresis (Heidelberg, Germany). *Salmonella* Typhimurium (ATCC 14028, as a Gram-negative bacterium), *Bacillus subtilis* (RCMB 010067, as a Gram-positive bacterium) and *Aspergillus fumigatus* (RCMB 02568, as a fungus) were provided by The Regional Center for Mycology and Biotechnology (Azhar University, Egypt).

### 2.2. Synthesis of *N*-quaternized (trimethyl) chitosan chloride

Trimethyl chitosan chloride was synthesized as reported in the literature.^25,26^ Briefly, 5 g Ch was suspended in a mixture of 80 mL of (CH_3_)_2_SO_4_ and 20 mL of dH_2_O under continuous stirring for 15 min.

**Fig. 2:**
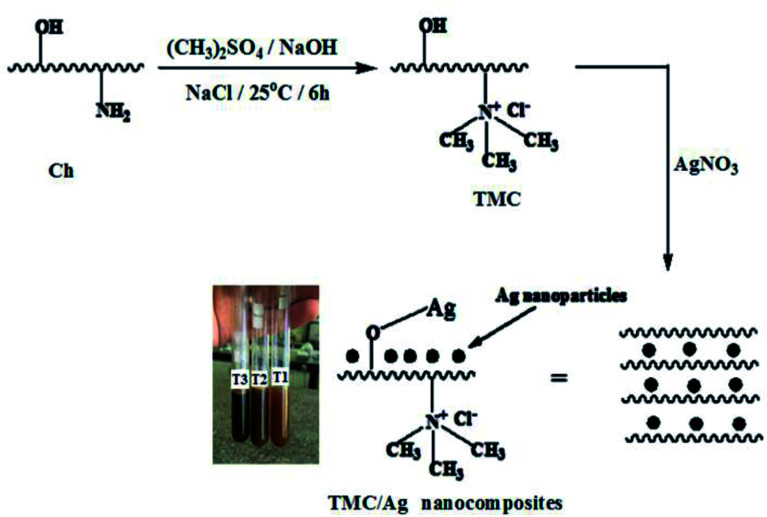
Scheme of the preparation of TMC/Ag, where T1, T2, and T3 are the TMC/Ag nanocomposites prepared using different AgNO_3_ concentrations (1, 2, and 3 mM), respectively.

In addition, NaOH (2.4 g) and NaCl (1.78 g) were added to the above Ch solution during a 10 minutes period, and then stirred well at 25 °C for 6 h. After 6 h, the TMC product was purified using dialysis tubes in dH_2_O for 3 days. Finally, TMC was precipitated in cold acetone (800 mL), collected by filtration, and then dried at 50 °C (Fig. 2).

### 2.3. Synthesis of nanocomposites

TMC (0.5 g) was dissolved in 50 mL dH_2_O in a round-bottomed flask (100 mL). Different concentrations of AgNO_3_ solution (1, 2 and 3) mM were slowly added in the dark to the above solution under continuous stirring at 25 °C overnight to form TMC/Ag nanocomposites, which were denoted as T1, T2 and T3, respectively (Fig. 2). The TMC solutions color changed from a pale yellow color (without AgNO_3_) to pale brown in the case of T1 (1 mM) and to a brown color for T2 (2 mM), and then to a dark brown color for T3 after the addition of AgNO_3_ (3 mM), which indicated the reduction of Ag^+^ to AgNPs (Ag^0^) inside the TMC chains.

### 2.4. Antimicrobial assay

The antimicrobial activity of the TMC and TMC/Ag nanocomposites was examined against foodborne pathogens such as *Bacillus subtilis* and *Salmonella* Typhimurium as Gram-positive and Gram-negative bacteria, respectively, and against *Aspergillus fumigates* as a fungus using the well diffusion Agar method in the presence of Sabouraud dextrose and nutrient agar media for both antifungal and antibacterial activities, respectively.^27^ Standard drugs such as amphotericin B and ampicillin were applied for comparison of the antifungal and antibacterial activities, respectively.

Briefly, 5 mL of sterilized medium was casted onto sterilized-Petri dishes and allowed to solidify. The diameter of the wells was 6 mm and 1 mg mL^−1^ of standard drugs (ampicillin and amphotericin B), TMC and the nanocomposites were added to each well using water as a control solvent. For antibacterial activity, the plates were incubated at 37 °C for 24 h. However, the plates were incubated for 48 h at 25 °C for antifungal activity. After the incubation time, the inhibition zone diameters of the tested samples and standard drugs were measured, and the mean value was taken for three reproducible experiments.

The minimum inhibitory concentration (MIC) is the minimum concentration of antimicrobial agent that inhibits the growth of pathogens through incubation overnight. MIC was determined using the micro dilution method in 96-well micro-plates for TMC and compared with that of the nanocomposites, which had higher inhibition zone diameters.

The stock solution of tested sample (1000 μg mL^−1^) was prepared using dH_2_O as the solvent, and then added to the plates. In addition, 100 μL of sterile broth was included in the well between row B and row H, and 100 μL of stock solution of the tested samples was added to the wells from rows A and B. Sterile broth and sample mixture in row B were added to each well to gain a two-fold serial dilution. Finally, the plates were incubated at 37 °C for 24 h for antibacterial activity, while incubation was performed at 25 °C for 48 h for antifungal activity.

The growth of the microorganisms was evaluated based on the turbidity and pellets at the bottom of the well. Optical density was evaluated using an ELISA reader (Vmax Molecular Devices, California, United States) at *λ* = 520 nm.^28,29^

### 2.5. Characterization

#### 2.5.1. Proton nuclear magnetic resonance (^1^H-NMR) spectroscopy

^1^H-NMR spectra of Ch and TMC were investigated using a Varian Mercury (VX-300 NMR) spectrometer (Bruker, Massachusetts, United States) at 300 MHz in dimethyl sulphoxide/trifluoroacetic acid as a control solvent for the Ch sample, while D_2_O was the control solvent for TMC.

#### 2.5.2. Fourier transform infrared (FTIR) spectroscopy

The FTIR spectra of the tested samples were measured on a JR-Affinity^−1^ Shimadzu Fourier transform spectrophotometer, Kyoto, Japan using KBr pellets in the wavenumber range of 4000 to 400 cm^−1^ at 25 °C.

#### 2.5.3. UV-Vis spectrophotometry

The absorption UV-Vis spectra of the Ag nanocomposites were measured using a Shimadzu (UV-Vis-NIR) spectrophotometer (Kyoto, Japan) in the wavelength range of 200 to 800 nm.

#### 2.5.4. High-resolution transmission electron microscopy (HR-TEM)

The diameter of the AgNPs was examined using an FEI-TEM, Netherland, with a dispersion of AgNPs in dH_2_O, which was obtained using a sonication bath for 10 min. Then, a drop of the solution was placed on a carbon-coated grid and dried for 30 min at 80 °C.

#### 2.5.5. X-ray diffraction (XRD)

The diffraction peaks of the examined samples were examined with an X-ray diffractometer (Philips X'pert MPD Pro, Malvern Panalytical, Malvern, United Kingdom) in the presence of an Ni-filter and Cu K_α_ radiation source at an accelerating voltage of 50 kV and current of 40 mA. The examined intensity was determined in the 2*θ* range of 5° and 60°.

#### 2.5.6. Field-emission scanning electron microscopy (FE-SEM)

Micro-images of the investigated samples were employed to examine their surface morphology using an FE-SEM (Quanta 250 FEG, FEI Company, Netherlands).

#### 2.5.7. Energy dispersive X-ray (EDX)

The elemental composition of the TMC and TMC/Ag nanocomposites was investigated using an EDX unit linked to an FE-SEM instrument.

#### 2.5.8. Statistical analysis

A statistical software package (SPSSR software, Version 16, SPSS Inc., Chicago, IL) was used to analyze the data with one-way analysis of variance (ANOVA).

## 3. Results and discussion

### 3.1. ^1^H-NMR spectroscopy

**Fig. 3:**
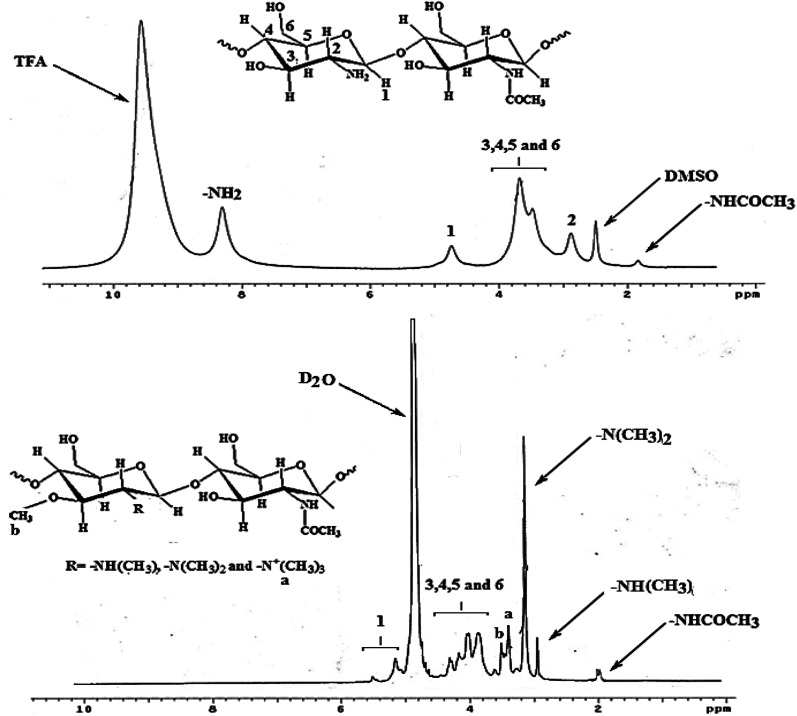
^1^H-NMR spectra of Ch and TMC.

The structure of TMC was elucidated using ^1^H-NMR spectroscopy and compared with the structure of Ch (Fig. 3). The spectrum of Ch was previously reported in the literature,^22,25^ which exhibits signals at *δ* = 1.8, 2.8, 4.7 and 8.3 ppm as singlets, which correspond to the *N*-acetylated group protons, glucosamine and its *N*-acetylated protons attached to the second carbon atom, anomeric proton (H_1_) and amino group protons, respectively. Additionally, signals appear at *δ* = 3.5–3.8 ppm as multiples, which correspond to the protons attached to the carbon atom no. 3–6.^30–33^

Furthermore, two new signals for DMSO and TFA, as solvents, appeared at *δ* = 2.5 and 9.6 ppm, respectively. However, the spectrum of TMC showed singlet signals at *δ* = 1.95, 2.9, 3.1, 3.35 and 3.47 ppm, corresponding to the *N*-acetylated group protons, protons of the *N*-methylated amino (–NH(CH_3_), –N(CH_3_)_2_, and –N^+^(CH_3_)_3_) groups, and protons of the methylated groups attached to the oxygen atom attached to carbon atom no. 3, respectively.^26,33^

Moreover, multiplet signals appeared at *δ* = 3.8–4.3 and 5.1–5.5 ppm, which are related to the protons attached to carbon atom no. 3–6 and the anomeric proton, respectively.^26,33,34^

In addition, the singlet signal corresponding to the –NH_2_ groups at *δ* = 8.3 ppm in the spectrum of Ch disappeared in the spectrum of TMC, which has no residual –NH_2_ groups. Furthermore, a sharp singlet signal appeared at *δ* = 4.8 ppm, corresponding to the D_2_O solvent for TMC. The *N*-quaternization degree (DQ)% for TMC was calculated to be 30% using the following equation:^33^DQ% = [(–N^+^(CH_3_)_3_)/9[H_1_]] × 100where –N^+^(CH_3_)_3_ refers to the integral area below the signal at *δ* = 3.35 ppm, while [H_1_] refers to the integral area below the signal at *δ* = 5.1–5.5 ppm.

### 3.2. FTIR spectroscopy

**Fig. 4:**
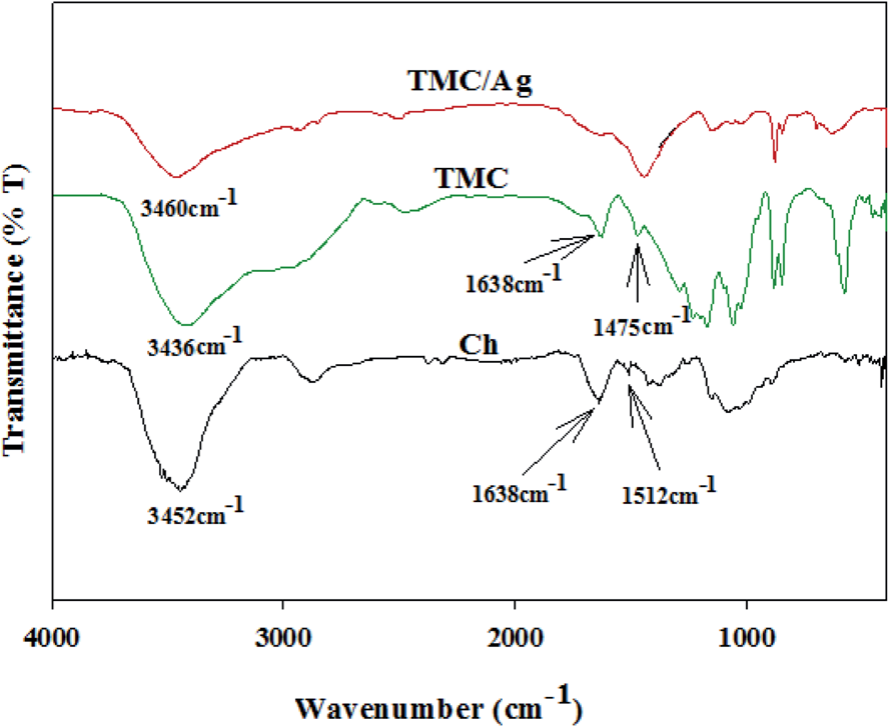
FTIR spectra of Ch, TMC and TMC/Ag nanocomposites.

The absorption peaks of the examined samples (TMC, Ch, and Ag nanocomposite) were observed in their FTIR spectra (Fig. 4). The spectrum of Ch illustrated peaks at 3452, 2874, 1638, 1512, 1150 and 895, 1080 and 1033 cm^−1^, corresponding to the hydroxyl (–OH) and amino (–NH_2_) groups, aliphatic (–C–H) groups, stretching vibration of the carbonyl (–C=O) groups, –NH_2_ groups, glycosidic (C–O–C) bonds in the repeating unit as the bending vibration of Ch, and 2^ry^ and 1^ry^ alcohol (C–OH) group stretching vibrations on the chains, respectively.^25,26,35,36^

However, some modifications were illustrated in the spectrum of TMC, such as a broad peak was observed at 3436 cm^−1^, corresponding to the stretching vibrations of both the –OH and –NH_2_ groups but with a lower intensity than that in the spectrum of Ch due to the conversion of the –NH_2_ groups to *N*-methylated amino groups (–NH(CH_3_), –N(CH_3_)_2_ and –N^+^(CH_3_)_3_). Moreover, an absorption peak appeared at 1638 cm^−1^, corresponding to –C=O groups. In addition, the peak for the –NH_2_ groups disappeared as the amino groups were converted to *N*-methyl amino groups. A new peak appeared at 1475 cm^−1^ in the spectrum of TMC, corresponding to *N*-methylated amino groups. However, the peak for the 2nd alcohol group in the Ch chains shifted between 1080 and 1057 cm^−1^, evidencing that *O*-methylation occurred only on the 2^ry^ –OH groups in TMC.^26^

Alternatively, the spectra of the TMC/Ag nanocomposites exhibited absorption peaks at 3460, 1638, 1057 and 1030 cm^−1^, corresponding to –OH, carbonyl, 2^ry^ alcohol and 1^ry^ alcohol groups as stretching vibrations, respectively. These peaks had a lower intensity than that observed in the spectrum of TMC due to the formation of coordination bonds between the Ag^+^ ions and oxygen atoms found in both the –OH and –C=O groups. Besides, another peak appeared at 700 cm^−1^, which is related to O–Ag.^37^

### 3.3. UV-Vis spectrophotometry

**Fig. 5:**
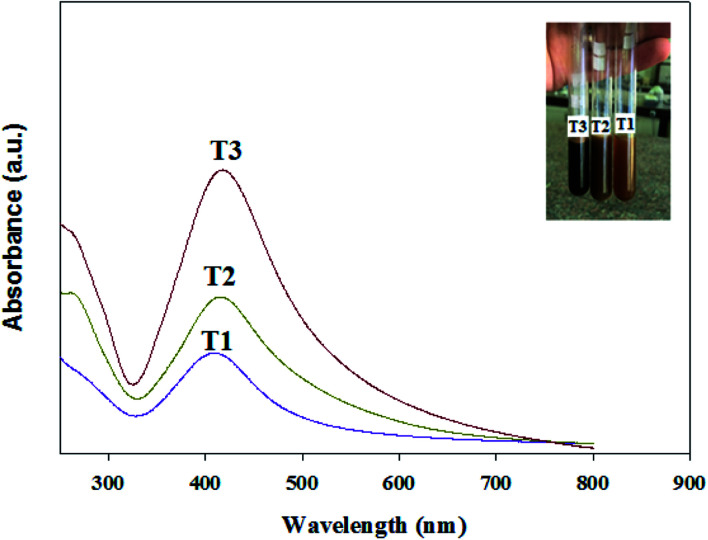
UV-Vis absorption spectra of the synthesized TMC/Ag nanocomposites with different concentrations of Ag nanoparticles (T1, T2 and T3).

The UV-Vis spectra of the Ag/TMC nanocomposites with different AgNP concentrations (T1, T2 and T3) are illustrated in Fig. 5. The spectra exhibit three peaks at 400, 410, and 415 nm for T1, T2 and T3, respectively, corresponding to the surface plasmon resonance of Ag^0^.^2,37^ The color of the TMC/Ag nanocomposite changed from a pale brown color (T1) to dark brown color (T3) due to the increase in the size of the nanoparticles. This observation was supported by the HR-TEM analysis.

### 3.4. HR-TEM analysis

**Fig. 6:**
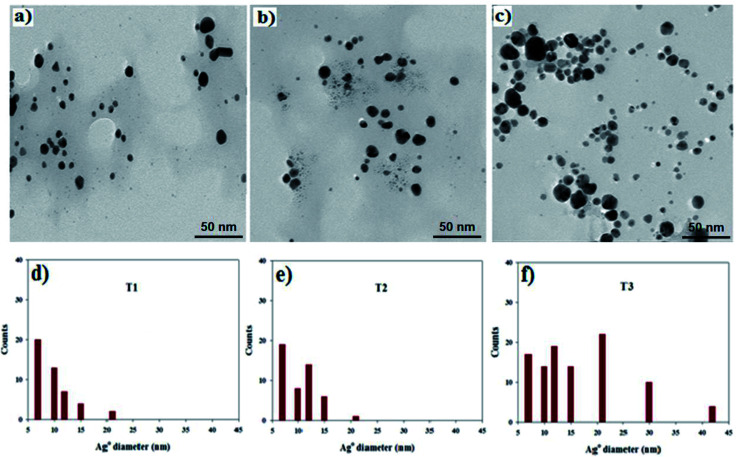
HR-TEM images of TMC/Ag nanocomposites with different AgNO_3_ concentrations. (a) 1 mM (T1), (b) 2 mM (T2) and (c) 3 mM (T3) and histograms of the nanoparticle size distribution for (d) T1, (e) T2 and (f) T3.

The Ag nanoparticles were synthesized using TMC as both a reducing and stabilizing agent using three various concentrations of AgNO_3_ of 1 mM (T1), 2 mM (T2) and 3 mM (T3). The TEM images of the Ag nanoparticles are shown in Fig. 6a–c, respectively. The Ag nanoparticles exhibited a good distribution as nanosized polydispersed spheres in the polymeric matrix. The histograms of the Ag nanoparticle size distribution are shown in Fig. 6d–f and the data illustrates that the Ag nanoparticles were synthesized with three particle size ranges (7–10, 12–15 and 21–42) nm, respectively. A small nanoparticle diameter range (7–10 nm) dominated in the TMC/Ag nanocomposite with a low AgNO_3_ concentration (T1), while a moderate particle diameter range (12–15 nm) dominated in the TMC/Ag nanocomposite with an AgNO_3_ concentration of 2 mM (T2). However, a large diameter range (21–42 nm) dominated in the TMC/Ag nanocomposites with an AgNO_3_ concentration of 3 mM (T3).

### 3.5. XRD analysis

**Fig. 7:**
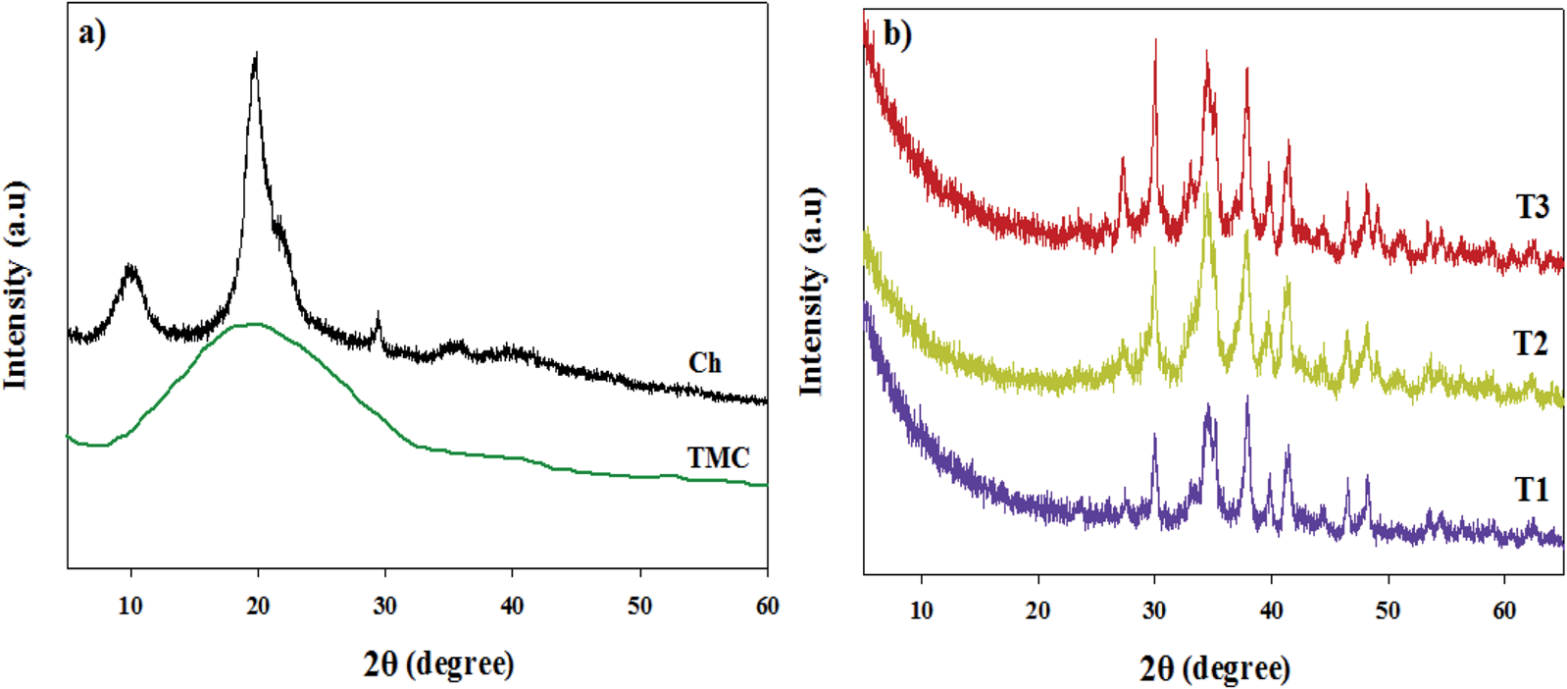
XRD patterns of (a) Ch and TMC and (b) TMC/Ag nanocomposites.

The XRD patterns of the tested samples, *i.e.*, Ch, TMC and TMC/Ag nanocomposites (T1, T2 and T3), are showed in Fig. 7a and b. The pattern of Ch (Fig. 7a) showed three peaks at 2*θ* = 9.5°, 20.0° and 30.0° due to the hydrogen bonding interactions between the Ch chains.^22,38^

In contrast, the pattern of TMC showed only one broad diffraction peak at 2*θ* = 20.0° (Fig. 7a). The formation of *N*-methylated groups on the Ch backbone led to the destruction of its crystallinity, resulting in a decrease in the H-bonding interactions among the TMC chains.

Alternatively, the XRD patterns of the nanocomposites (Fig. 7b) showed that the broad peak at 2*θ* = 20.0° for TMC disappeared, which is due to the destruction of the interactions between the TMC chains *via* the *in situ* formation of AgNPs in the TMC chains. Also, the nanocomposites showed four sharp diffraction bands at 2*θ* = 30°, 35°, 38°, and 42° and four small diffraction bands at 2*θ* = 28°, 40°, 46°, and 48°. The intensity of these diffraction bands increased with an increase in the concentration of Ag, which corresponds to the crystalline nature of the in situ-prepared AgNPs. This is similar to the *in situ* preparation of AgNPs in the presence of bagasse and modified chitosan/rectorite, which was reported in the literature.^39,40^

### 3.6. FE-SEM and EDX analysis

**Fig. 8:**
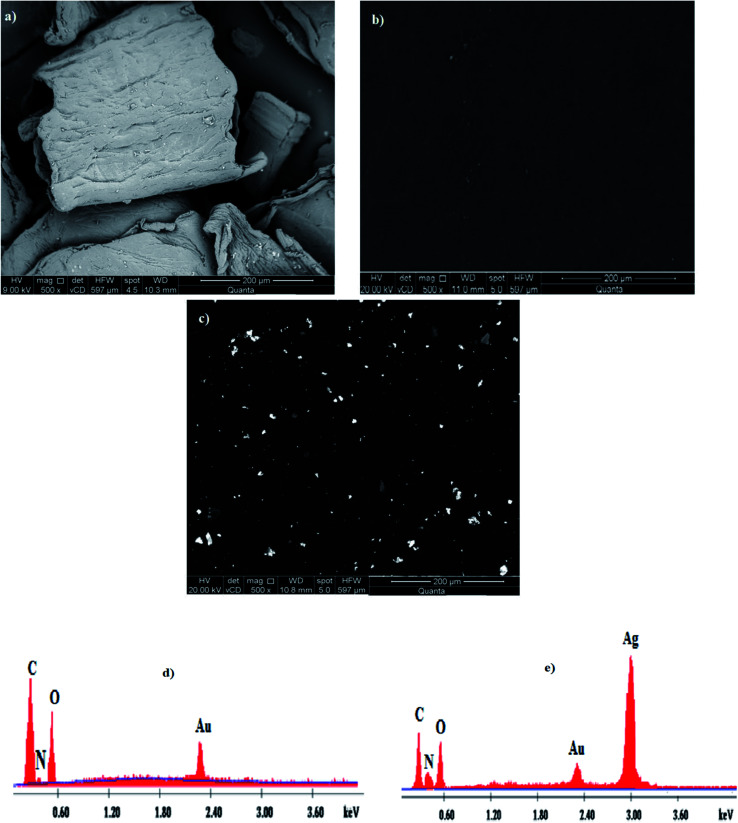
FE-SEM images of (a) Ch, (b) TMC and (c) TMC/Ag nanocomposite (magnification ×500) with EDX spectra of (d) TMC and (e) TMC/Ag nanocomposite.

The FE-SEM images of the examined samples, *i.e.*, Ch, TMC and TMC/Ag nanocomposite, showed their surface morphology with a magnification of ×500, as shown in Fig. 8a–c, respectively, showing enormous surface changes for all the samples. The surface morphology of Ch was reported previously in the literature^25^ to appear as lobules having a regular crystalline structure, resulting from the hydrogen bonding interactions among its chains. However, TMC showed a smooth surface because bulky *N*-methylated groups were formed, which led to the destruction of the interactions among the Ch chains.^25,41^ In contrast, the surface of the TMC/Ag nanocomposites revealed the formation of spherical bead-like AgNPs on the compact surface of TMC. Consequently, the surface of the TMC/Ag nanocomposites showed the successful synthesis of Ag nanoparticles in the presence of TMC as a self-reducing and stabilizing agent.

Moreover, the elemental composition of the TMC and Ag nanocomposites was determined *via* an EDX unit linked to an FE-SEM instrument. The data, as shown in Fig. 8d and e, showed three elemental peaks for TMC at 0.27, 0.40, and 0.56 keV, corresponding to the C, N, and O elements, respectively. The spectra of the TMC/Ag nanocomposites showed four peaks at 0.27, 0.42, 0.58 and 3.0 keV, which are assigned to the C, N, O, and Ag elements, respectively. The appearance of a peak for silver in the spectra of the TMC/Ag nanocomposites proved the successful synthesis of Ag nanoparticles inside the TMC chains.

### 3.7. Antimicrobial assay

**Fig. 9:**
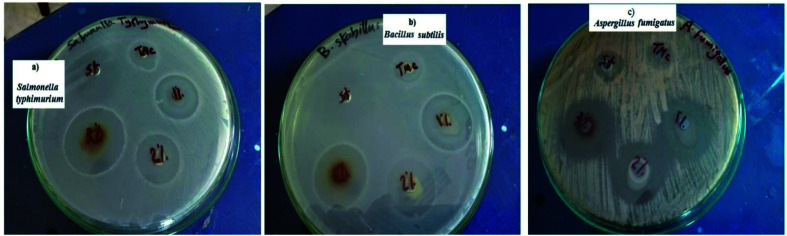
Antimicrobial activity of TMC and TMC/Ag nanocomposites with different Ag nanoparticle contents compared to the reference drugs against foodborne pathogens. (a) *Salmonella* Typhimurium, (b) *Bacillus subtilis*, and (c) *Aspergillus fumigatus*.

The antimicrobial activity results are tabulated in Table 1, while the inhibition zones for the TMC and Ag nanocomposites are shown in Fig. 9, which reveal that TMC had higher antimicrobial activity compared to the reference drugs against the three foodborne pathogens with inhibition zone diameters of 21.3, 16.7, and 20.0 mm against *S.* Typhimurium, *B. subtilis* and *A. fumigates*, respectively. However, the TMC/Ag nanocomposites exhibited higher inhibition zone diameters against *S.* Typhimurium of 30.7 mm (T1), 33.7 mm (T2), and 37.0 mm (T3), 26.0 mm (T1), 26.0 mm (T2), and 32.7 mm (T3) against *B. subtilis*, and 29.0 mm (T1), 29.8 mm (T2), and 30.0 mm (T3) against *A. fumigates*.

**Table 1:** Antimicrobial activity of TMC and TMC/Ag nanocomposites (T1, T2 and T3) compared to reference drugs against foodborne pathogens

Sample code	Diameter of inhibition zone (mm)		
	Gram-negative bacterium	Gram-positive bacterium	Fungus
	*S. typhimurium*	*B. subtilis*	*A. fumigates*
Ampicillin (reference)	33.7 ± 1.53^a^	26.0 ± 1.0^a^	—
Amphotericin B (reference)	—	—	21.0 ± 1.0
TMC	21.3 ± 1.53	16.67 ± 0.88	20.0 ± 0.66^a^
TMC/Ag (T1)	30.7 ± 0.66^a^	26.0 ± 0.66^a^	29.0 ± 0.88^a^
TMC/Ag (T2)	33.7 ± 0.72^a^	26.0 ± 0.22^a^	29.8 ± 0.38^a^
TMC/Ag (T3)	37.0 ± 1.0^a^	32.67 ± 1.05^a^	30.0 ± 0.72^a^

a There is a significant difference at *p*-value < 0.05.

Several mechanisms to explain the antibacterial activities of Ch have been reported, where among them, the most acceptable one is that based on the ionic force interactions between –^+^NH_3_ on Ch and negative charges on the bacterial cell surface, especially the cytoplasmic membrane,^42–44^ while the exact mechanism of the antifungal activity of Ch remains controversial. However, the proposed mechanism for the antifungal activity of Ch is also the electrostatic force interactions between the cationic groups on the Ch chains and negative charges on the cell surface, leading to a decrease in intracellular electrolytes and proteinaceous constituents.^45^ The antimicrobial activity of TMC (DQ was 30%) was due to the ionic interaction between the protonated –^+^NH_2_CH_3_, –^+^NH(CH_3_)_2_, and –^+^N(CH_3_)_3_ groups on Ch and the electronegative charges on the bacterial and fungal cell surfaces. Also, the data exhibited that the TMC/Ag nanocomposites had higher antimicrobial activity than the unmodified TMC, which increased with an increase in the concentration of Ag (T3 had the highest antimicrobial activity).

Ag has good antimicrobial activity given that it destroys the cell membrane of microorganisms through its interactions with the phosphorous and sulfur elements present in the cell membrane, which leads to a disturbance in its functions.^7,37^ An increase in the concentration of AgNPs led to an increase in antimicrobial activity for the TMC/Ag nanocomposites because of the increase in the surface area of the Ag nanoparticles, which enhances the interactions with the cell wall of microorganisms.

The statistical analysis of the antimicrobial activity data revealed significant differences (*p*-value < 0.05) for the TMC/Ag nanocomposites, which were compared with both TMC and reference drugs (Table 1). Additionally, an insignificant variance was observed between TMC and the reference drugs against *S.* Typhimurium and *B. subtilis* (*p*-value > 0.05).

The MIC was examined for TMC and the TMC/Ag nanocomposite with the highest Ag content (T3) and compared with that of reference drugs (ampicillin and amphotericin B) against foodborne pathogens using the brain heart infusion broth micro dilution method and the data is tabulated in Table 2. The MIC for TMC was 125, 62.5, and 7.81 μg mL^−1^, while that of the TMC/Ag nanocomposite was 0.25, 0.49, and 0.49 μg mL^−1^ against *S.* Typhimurium, *B. subtilis*, and *A. fumigates*, respectively. The MIC results for ampicillin were 0.49 and 0.98 μg mL^−1^ against *S.* Typhimurium and *B. subtilis*, respectively, and that of amphotericin B was 3.90 μg mL^−1^ against *A. fumigates*. The data showed that the TMC/Ag nanocomposite (T3) had greater antimicrobial activity against foodborne pathogens compared to both reference drugs and TMC individually. Notably, the antibacterial results of the prepared TMC/Ag nanocomposite were higher than that in other reported works such as chitosan/Ag nanocomposite^46^ and chitosan biguanidine-grafted poly(3-hydroxybutyrate) copolymer/Ag nanocomposite^47^ against the growth of *S.* Typhimurium and *A. fumigates*, and magnetic cellulose/Ag nanocomposite^48^ and chitosan biguanidine hydrochloride/Ag nanocomposite^30^ against the growth of *B. subtilis*.

**Table 2:** Minimum inhibitory concentration (MIC) of TMC and TMC/Ag nanocomposite (T3) compared with reference drugs against foodborne pathogens

Sample code	MIC (μg mL^−1^)		
	Gram-negative bacterium	Gram-positive bacterium	Fungus
	*S. typhimurium*	*B. subtilis*	*A. fumigates*
Ampicillin (reference)	0.49	0.98	—
Amphotericin B (reference)	—	—	3.90
TMC	125	62.5	7.81
TMC/Ag (T3)	0.25	0.49	0.49

## 4. Conclusions

The one-pot green synthesis of TMC/Ag nanocomposites was successfully performed using TMC as both a self-reducing and stabilizing agent with different AgNO_3_ concentrations of 1 mM, 2 mM, and 3 mM. The structure of the TMC/Ag nanocomposites was analyzed using different characterization techniques including FTIR, UV-Vis, XRD, and FE-SEM/EDX. The obtained spectra supported the successful *in situ* preparation of Ag NPs in TMC. Furthermore, the HR-TEM images and their histograms showed the good distribution of AgNPs in the polymeric matrix as polydispersed nano-spheres and the XRD patterns exhibited that the AgNPs has good crystallinity. Additionally, the antimicrobial assay results revealed the highest antimicrobial activity for the Ag nanocomposites against foodborne pathogens including *B. subtilis* and *S.* Typhimurium as Gram-positive and Gram-negative bacteria, respectively. The antimicrobial assay results revealed the highest antifungal activity for the Ag nanocomposites against *A. fumigates* compared with both TMC and the reference drugs, which increased with an increase in the concentration of Ag nanoparticles in the polymeric matrix.

## Conflicts of interest

The authors declare no conflict of interest.

## Supplementary Material
